# Reliability and Accuracy of the Fitbit Charge 4 Photoplethysmography Heart Rate Sensor in Ecological Conditions: Validation Study

**DOI:** 10.2196/54871

**Published:** 2025-01-08

**Authors:** Maxime Ceugniez, Hervé Devanne, Eric Hermand

**Affiliations:** 1 ULR 7369 - URePSSS - Unité de Recherche Pluridisciplinaire Sport Santé Société, Univ. Littoral Côte d’Opale, Univ. Lille, Univ. Artois, 189b, Avenue Maurice Schumann, Centre Universitaire des Darses, Dunkerque, 59375, France, 33 328237357; 2 UMR INSERM U1272 Hypoxie & Poumon, Département STAPS, Université Sorbonne Paris Nord, Bobigny, France

**Keywords:** photoplethysmography, physical activity, ecological conditions, accuracy, reliability, Fitbit Charge 4, Fitbit, exercise, ecological, wrist-worn device, device, sensor, wearables, usefulness, variability, sensitivity, heart rate, heart rate sensor

## Abstract

**Background:**

Wrist-worn photoplethysmography (PPG) sensors allow for continuous heart rate (HR) measurement without the inconveniences of wearing a chest belt. Although green light PPG technology reduces HR measurement motion artifacts, only a limited number of studies have investigated the reliability and accuracy of wearables in non–laboratory-controlled conditions with actual specific and various physical activity movements.

**Objective:**

The purpose of this study was to (1) assess the reliability and accuracy of the PPG-based HR sensor of the Fitbit Charge 4 (FC4) in ecological conditions and (2) quantify the potential variability caused by the nature of activities.

**Methods:**

We collected HR data from participants who performed badminton, tennis, orienteering running, running, cycling, and soccer while simultaneously wearing the FC4 and the Polar H10 chest belt (criterion sensor). Skin tone was assessed with the Fitzpatrick Skin Scale. Once data from the FC4 and criterion data were synchronized, accuracy and reliability analyses were performed, using intraclass correlation coefficients (ICCs), Lin concordance correlation coefficients (CCCs), mean absolute percentage errors (MAPEs), and Bland-Altman tests. A linear univariate model was also used to evaluate the effect of skin tone on bias. All analyses were stratified by activity and pooled activity types (racket sports and running sports).

**Results:**

A total of 77.5 hours of HR recordings from 26 participants (age: mean 21.1, SD 5.8 years) were analyzed. The highest reliability was found for running sports, with ICCs and CCCs of 0.90 and 0.99 for running and 0.80 and 0.93 for orienteering running, respectively, whereas the ICCs and CCCs were 0.37 and 0.78, 0.42 and 0.88, 0.65 and 0.97, and 0.49 and 0.81 for badminton, tennis, cycling, and soccer, respectively. We found the highest accuracy for running (bias: 0.1 beats per minute [bpm]; MAPE 1.2%, SD 4.6%) and the lowest for badminton (bias: −16.5 bpm; MAPE 16.2%, SD 14.4%) and soccer (bias: −16.5 bpm; MAPE 17.5%, SD 20.8%). Limit of agreement (LOA) width and artifact rate followed the same trend. No effect of skin tone was observed on bias.

**Conclusions:**

LOA width, bias, and MAPE results found for racket sports and soccer suggest a high sensitivity to motion artifacts for activities that involve “sharp” and random arm movements. In this study, we did not measure arm motion, which limits our results. However, whereas individuals might benefit from using the FC4 for casual training in aerobic sports, we cannot recommend the use of the FC4 for specific purposes requiring high reliability and accuracy, such as research purposes.

## Introduction

Over the recent years, connected bracelet and watch sales have been regularly increasing [1]. These tools allow individuals to monitor various active life parameters, such as time of activity, sleep quality, step numbers, and energy expenditure, among others. Most of them are computed through algorithms that use accelerometry and heart rate (HR) data. Although these new devices are used for training by both recreational athletes and elite athletes, chest belts remain the gold standard, especially among high-level athletes, for measuring HR, as they are based on R peak detection from the QRS electrocardiogram (ECG) complex [2,3]. In opposition to chest belts, other wearables perform additional measurements (eg, accelerometry and positioning measurements via a GPS), which, when combined with HR data and algorithm processing, provide data on other parameters, such as energy expenditure and quality of sleep. Moreover, wrist-worn devices could reduce the tolerance and acceptability issues observed with chest belts [4]. Wrist-worn devices usually estimate continuous HR through the photoplethysmography (PPG) technique, which was first used in the late 1930s [5]. PPG involves measuring light absorption through tissues of interest [6]; red and infrared lights are emitted by an LED through the skin, and a photoreceptor captures the remaining emissions after tissue absorption [7]. However, even if the concept remains similar, connected watches usually come with a green light PPG sensor for its ability to reduce motion artifacts, contrary to the red ones commonly used in the medical field for blood oxygen saturation evaluation [8,9]. The reason for this is that the deeper the light penetrates the tissue (eg, red wavelength), the more the pulse wave is affected by limb movements [10,11]. As light penetration depends on light wavelength, the shorter wavelength of green light provides less information from deeper nonpulsatile tissues [9,12]. Considering this, green light is less prone to motion artifacts during normal daily life [13-15]. In the case of HR monitoring via watches or bracelets during activities, signal accuracy and reliability may vary according to numerous factors. Among them, gear placement on skin, strap tightening (which induces skin compression), skin tone, and activity type and intensity can affect HR recording [15-21]. PPG HR sensor accuracy has been investigated during physical activity across a spectrum of intensities. However, researchers tend to measure PPG HR sensor accuracy with treadmill running or cycling ergometers in laboratory-controlled conditions [17,22-24]. To our knowledge, only a few evaluations of connected device accuracy (eg, accuracy of a smartwatch, as evaluated in this paper) were performed in ecological conditions across different physical activity types. As this type of device is meant to be used in non–laboratory-controlled conditions or free-living conditions, this study aimed to evaluate the accuracy and validity of the PPG HR data from the Fitbit Charge 4 (FC4; Fitbit LLC) across multiple physical activity types. Therefore, the objectives of this study were to (1) assess the accuracy and reliability of FC4 HR measurement in ecological conditions and (2) quantify the potential impact of activity type on accuracy.

## Methods

### Participants

A total of 26 healthy young adults from the Sport Sciences University of Calais, France, who were practicing physical activities on a weekly basis, volunteered and were included in this study, which was advertised on the university campus and via social networks. No inclusion or exclusion criteria were used.

A minimum HR sample size was calculated with G*Power (version 3.1.9.6) [25] by using a significance level (α) of 5%, a statistical power of 1 – β = 80%, and an effect size of 0.075 (computed from the expected HR mean and SD). The number of necessary HR samples was below 6000, representing 100 minutes of recording (sample rate=1 s^−1^). Measurements were performed during participants’ regular training sessions for soccer, badminton, orienteering running, basketball, tennis, and road biking.

### Ethical Considerations

This study was approved by the National Commission for Data Protection and Liberties (CNIL-France; registration number: 2224247). All participants gave their written informed consent, with the possibility to opt out of the protocol at any point. Data collected throughout the protocol were deidentified for privacy and confidentiality reasons. Finally, although participants could not be financially compensated for their participation, which did not impact their usual routine, each of them personally received an individualized analysis of their HR data, so that they could receive information about cardiac demand during various phases of their training sessions (intensity levels, duration, and cardiac work zone) and adjust their sessions’ contents if needed.

### Data Collection

PPG HR signals from the FC4 were compared to those from the Polar H10 thoracic belt (Polar Electro Oy), which was used as the criterion sensor [26]. The assessment of skin tone was performed with the Fitzpatrick Skin Scale, which ranges from 1 (lightest tone) to 6 (darkest tone) [27].

Participants were asked to wear both sensors simultaneously at each session. The FC4 was placed on the wrist of the nondominant arm (ie, around 2 cm away [proximal] from the ulnar styloid process), whereas the Polar H10 thoracic belt was placed under the thorax (ie, on the xyphoid process) and paired with Polar V800 wristwatches for HR recording. Each device was placed firmly against the skin, as recommended by the manufacturer’s instructions.

### Data Extraction and Analysis

Data were extracted through both companies’ web services (ie, the Fitbit app [28] and Polar Flow website [29] for the FC4 and Polar H10, respectively). A MATLAB script (The MathWorks Inc) was then used to synchronize the two devices’ HR measurements. Record alignments were performed by using a least square method to minimize squared deviation between FC4 and Polar H10 records, and they were smoothed over a 10-second window to calibrate the sample rate from both devices, as previously described [18,30]. Data normality was verified by a Kolmogorov-Smirnov test.

FC4 artifact data were defined as values that deviated from the criterion data by 20 beats per minute (bpm). Bland-Altman tests were performed on smoothed data to assess the accuracy of FC4 HR data by participant and by activity [31]. Means (bias) and SDs of the differences between the FC4 and H10 values were used to evaluate upper and lower limits of agreement (LOAs), per the following formula: upper/lower LOA = bias ± 1.96 × SD. Mean absolute error (MAE) and mean absolute percentage error (MAPE) were calculated to quantify mean differences between FC4 and Polar H10 HR data. Two tests were performed to evaluate the reliability of the FC4: (1) 2-way random intraclass correlation coefficients (ICCs) with an absolute consistency type were calculated and interpreted according to current guidelines (ICC<0.5: poor; 0.5<ICC<0.75: moderate; 0.75<ICC<0.90: good; ICC>0.90: excellent reliability) [32], and (2) a computation of Lin concordance correlation coefficients (CCCs) was performed, interpreted following McBride’s [33] recommendations (CCC<0.90: poor; 0.90<CCC<0.95: moderate; 0.95<CCC<0.99: very good; CCC>0.99: almost perfect strength of agreement).

All statistical analyses were stratified by activity type, and a Kruskal-Wallis test was performed to compare activity bias, with activity types as independent groups. Mann-Whitney *U* tests were implemented to compare bias from 0 among activity types (independent groups). Further, a linear univariate model was used to estimate the effect of skin tone on bias while controlling the impact of activity type. Statistics were performed using IBM SPSS statistics 25 software (IBM Corp).

## Results

### Participants’ Characteristics

A total of 26 young adults (11 women and 15 men) were included in this study. Their characteristics are compiled in Table 1. In total, 77.5 hours of practice were recorded, distributed across 55 sessions (Table 2).

**Table 1.** Participants’ characteristics.

	Male (n=15), mean (SD)	Female (n=11), mean (SD)	All participants (N=26), mean (SD)
Age (y)	21.2 (7.0)	20.8 (3.7)	21.1 (5.8)
Weight (kg)	75.8 (9.6)	57.6 (8.9)	68.1 (12.9)
Height (cm)	183 (6)	166 (8)	176 (11)
BMI (kg/m^2^)	22.6 (1.8)	20.9 (1.7)	21.9 (1.9)
Skin tone (Fitzpatrick Skin Scale score)	2.9 (0.6)	2.8 (0.6)	2.9 (0.6)

**Table 2.** Descriptive data of recorded sessions.

Activity		Sessions, n	Recorded time, h	Participants, n
Racket sports				
	Badminton	10	15.07	7
	Tennis	3	4.90	2
	Total	13	19.96	9
Running sports				
	Orienteering running	5	7.02	5
	Run	11	14.09	3
	Total	16	21.10	8
Other sports				
	Bike	13	18.39	2
	Soccer	13	18.01	12

### Accuracy and Artifact Percentage

Biases, LOAs, and artifact percentages are shown in Table 3.

**Table 3.** Bland-Altman analyses, mean absolute error (MAE), and mean absolute percentage error (MAPE) by activity.

Activity		Bland-Altman analyses, bpm^a^			Artifact ratios, %	bpm, MAE (SD)	bpm, MAPE (SD)
		Bias	Upper LOA^b^; lower LOA	LOA width			
Racket sports							
	Badminton	−16.5	35.2; −68.2	103.5	39.3	21.7 (22.3)	16.2 (14.4)
	Tennis	−6.2	24.8; −37.2	62.0	22.7	12.8 (11.2)	8.9 (7.4)
	Total	−14.0	34.3; −62.3	96.6	35.2	19.5 (20.5)	14.4 (13.4)
Running sports							
	Orienteering running	−8.6	26.0; −43.3	69.4	17.0	11.7 (15.9)	9.5 (10.4)
	Run	0.1	10.9; −10.7	21.6	2.5	1.7 (5.2)	1.2 (4.6)
	Total	−2.8	20.5; −26.1	46.6	7.3	5.0 (11.1)	4.0 (8.1)
Other sports							
	Bike	4.8	36.8; −27.3	64.1	18.1	10.4 (10.4)	8.1 (11.0)
	Soccer	−16.5	26.7; −59.6	86.3	35.5	19.2 (19.7)	17.5 (20.8)

a bpm: beats per minute.

b LOA: limit of agreement.

**Figure 1.**
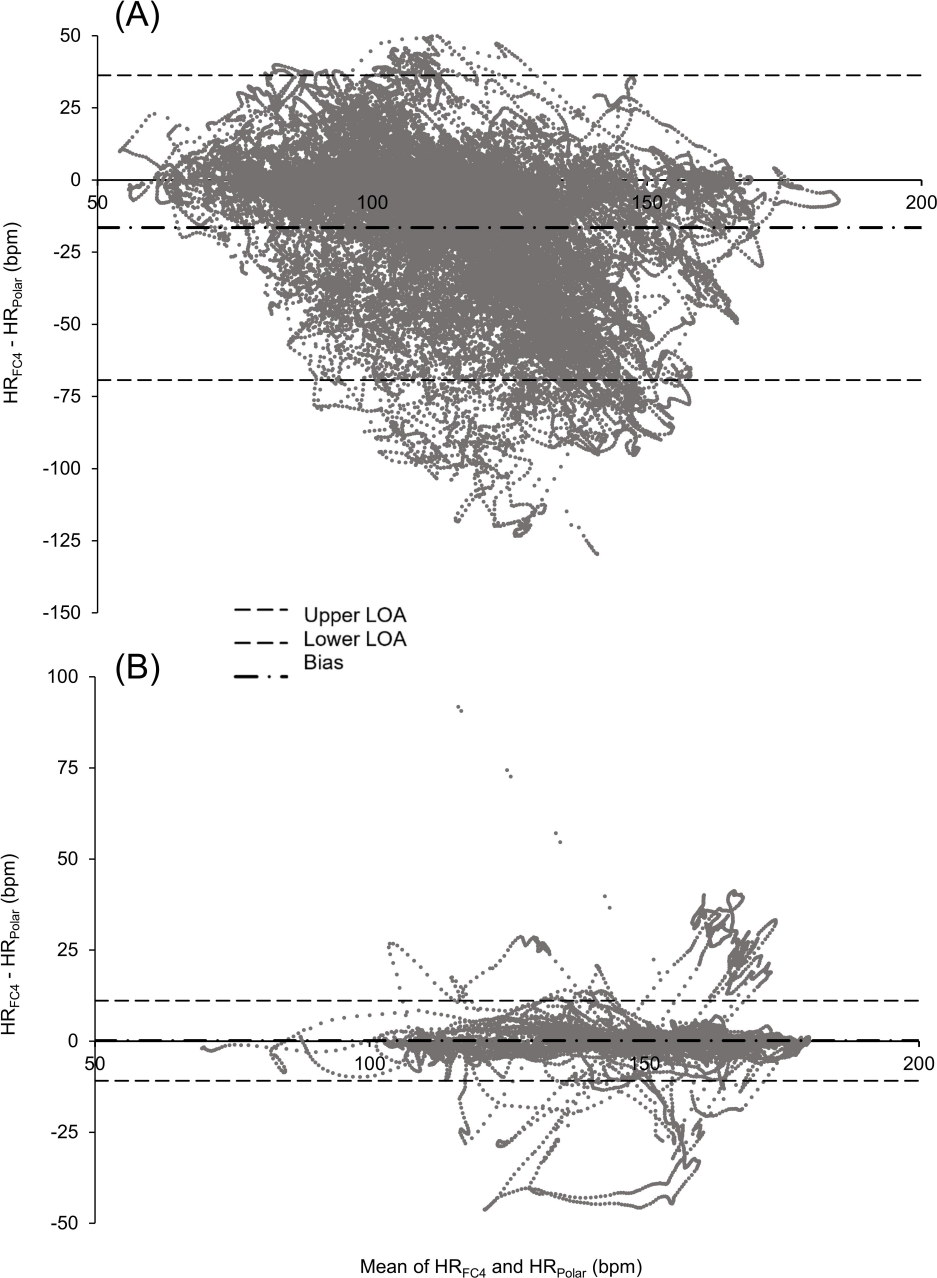
Bland-Altman plots for (A) badminton and (B) running. Badminton exhibits the highest bias, and the bias for running is the closest to the origin. Each activity represents, respectively, 14 and 15.07 hours of recording. bpm: beats per minute; FC4: Fitbit Charge 4; HR: heart rate; LOA: limit of agreement; Polar: Polar H10.

Biases were different between each activity (*P*<.001), with all of them also being different from 0 (*P*<.001). The lowest bias values, MAEs, and MAPEs were found for running and cycling, and the highest ones were found for badminton and soccer. Furthermore, the narrowest LOA width was found for running, and the widest was found for badminton (Figure 1). We found similar results by grouping activities; the lowest bias, MAE, MAPE, and LOA width were found for running activities (running and orienteering running), and the largest ones were found for racket sports (badminton and tennis; Table 3).

### Reliability

ICCs and CCCs are presented in Table 4. ICCs and CCCs indicated poor reliability (<0.50 and <0.90, respectively) for racket sports and soccer but excellent reliability for running overall (the total ICC and CCC for all running activities overall were >0.90 and >0.99, respectively). The largest percentages of artifact HR data were found for badminton and soccer, whereas running exhibited the lowest (Table 3). The results were similar when pooling activities; the highest rate of artifact HR data was found for racket sports, and the lowest was found for running sports (Table 3).

**Table 4.** Intraclass correlation coefficients (ICCs) and Lin concordance correlation coefficients (CCCs) by single and pooled activities.

Activity		ICC (95% CI)	CCC
Racket sports			
	Badminton	0.365 (0.517-0.154)	0.778
	Tennis	0.421 (0.320-0.421)	0.884
	Total	0.435 (0.237-0.574)	0.883
Running sports			
	Orienteering running	0.801 (0.865-0.801)	0.932
	Run	0.900 (0.898-0.900)	0.999
	Total	0.926 (0.915-0.935)	0.996
Other sports			
	Bike	0.658 (0.702-0.603)	0.971
	Soccer	0.487 (0.158-0.487)	0.809

### Effect of Skin Tone

Mean Fitzpatrick Skin Scale scores are shown in Table 1. No overall interaction and no interaction in each activity were found between bias and skin tone, while being standardized by activity type.

## Discussion

### Main Results

**Figure 2.**
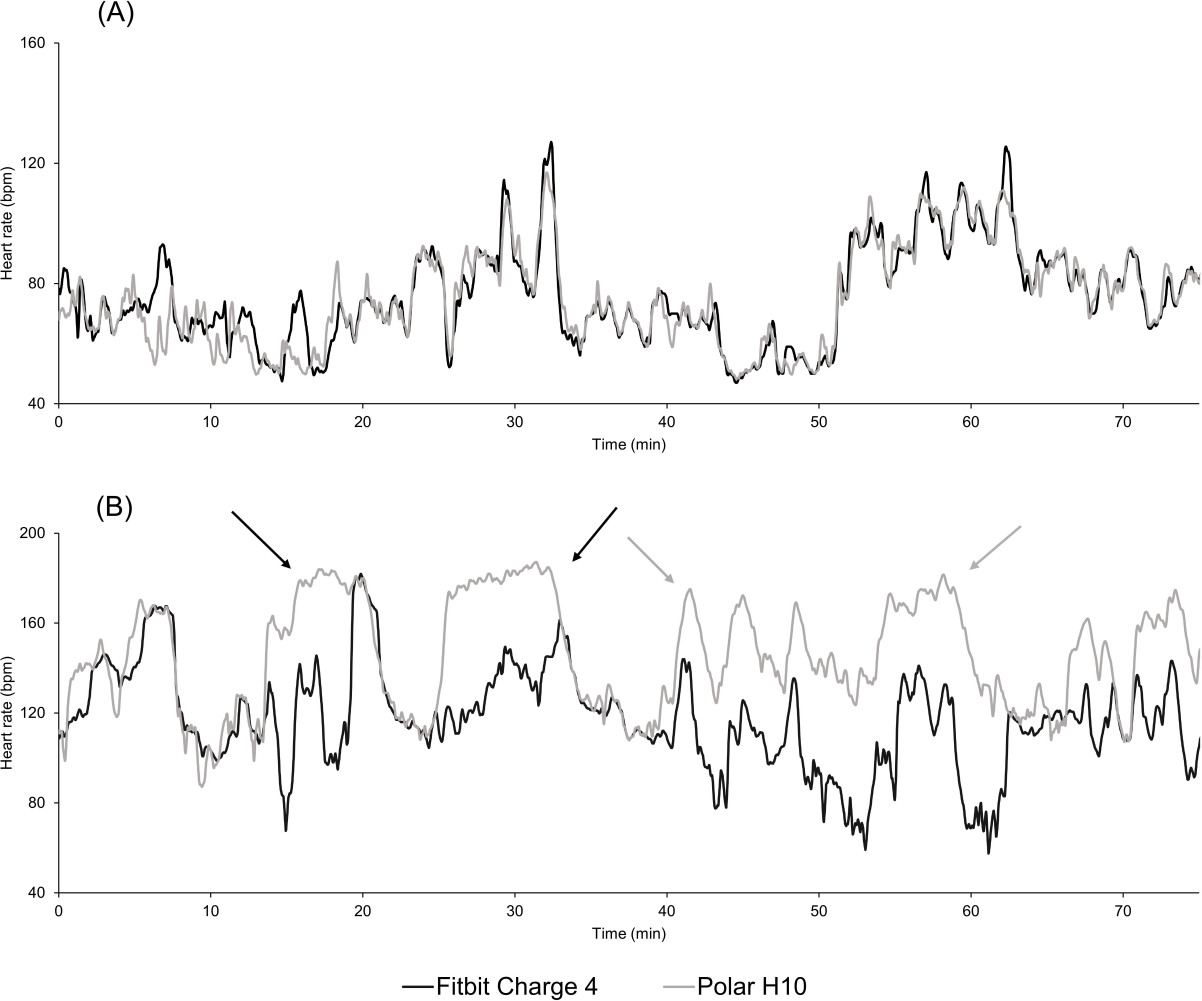
Examples of synchronized heart rate signals (Polar H10: light grey; Fitbit Charge 4: black; A: orienteering running; B: soccer). Soccer heart rate data show recurrent sudden uncoupling between Fitbit Charge 4 and Polar H10 data, as well as heart rate underevaluations by the Fitbit Charge 4 throughout the recording session. bpm: beats per minute.

In this study, we evaluated the HR accuracy and reliability of the FC4 by comparing it to the Polar H10 chest belt (criterion sensor) in non–laboratory-controlled conditions. Our results showed negative biases for most activities (except running and cycling; Table 3), the presence of artifact data, and HR underevaluation by the FC4 (Figure 2). These results are similar to earlier findings that show the tendency of PPG wrist sensors to overestimate or underestimate HR [34-36]. ICCs and CCCs were fluctuant, mainly depending on the activity type. The FC4 shows good reliability for running activities, with almost perfect and moderate CCCs and excellent and good ICCs for running and orienteering running, respectively. Additionally, running was the activity with the lowest bias, the lowest MAPE, and the smallest LOA width. On the other hand, we found lower ICCs for badminton, soccer, and cycling, which also showed higher artifact ratios. Thus, we suggest that the excessive amount of arm movement in these activities could affect HR recording, as previously shown [37]. Badminton and tennis are characterized by “sharp” movements and rotations of the nondominant arm, whereas soccer can induce some instability of the sensor due to random arm and wrist actions, which can result in watches sliding over skin and the transient loss of HR signals (Figure 2). Furthermore, although cycling shows an overall good CCC (0.971), the ICC (0.658), MAPE (8.1%, SD 11.0%), and LOA width (64.03 bpm) indicate a lack of reliability during this activity. Since cycling remains a lower limb cyclic activity, there are little to no arm movements that may affect sensor placement. However, wrist position on handlebars (eg, during road cycling) and the contractility of wrist muscle flexors and extensors could alter vascular arteriovenous system detection and lower signal quality while enhancing compression forces [6,8,16,17]. ICCs and CCCs were calculated for pooled activities—running sports (running and orienteering running) and racket sports (tennis and badminton). These activities mostly rely on the same corporal pattern, and grouping them allowed us to equilibrate the time of practice between other activities. Our data showed no differences in ICC or CCC parameters, with those for running sports and racket sports showing excellent and poor reliability, respectively (ICC: 0.926 vs 0.435; CCC: 0.996 vs 0.883; Table 4).

Overall, artifacts and random movements could highly affect a sensor’s precision. Some studies tried to reduce motion artifacts by using novel techniques, such as accelerometry coupling or algorithm-based processing, but there is no consensus yet on which one should be used [38-42]. Even by varying numbers of diodes, lights colors, and algorithms, the technical aspect for measuring HR remains similar, suggesting that some FC4 characteristics could increase the presence of motion artifacts. For example, the materials used for the strap was slippery on skin, amplified by exercise-induced sudation. In addition, from a general standpoint, algorithms used by manufacturers can also affect the recordings, but we did not have access to these proprietary processing scripts.

### Limitations

We observed no influence of skin tone on bias, unlike previous works that highlighted some effects on HR error rates [21]. However, our study is in line with another paper that was based on analyses of the signal to noise ratio, which showed no effect of skin tone when using a proper PPG wavelength (520 nm) [15]. More recently, another study did not find an effect of skin tone on beat-to-beat interval quality while separating skin types into two major groups (group 1: 1 to 4 on the Fitzpatrick Skin Scale; group 2: 5 and 6 on the Fitzpatrick Skin Scale) [43]. Even if our study findings are consistent with these results, no generalization can be made, since participants’ skin types ranged from 2 (n=6) to 4 (n=3) on the Fitzpatrick Skin Scale. Moreover, we did not take into account physiological and environment factors, such as local temperature, humidity, and sudation, which may impact peripheral vasomotricity and therefore increase or decrease PPG signal intensity [44-46]. It would also have been relevant to assess the influence of motion parameters, such as acceleration measured at the wrist, on HR accuracy and reliability of the FC4; however, we could not access raw data produced by the proprietary processes for further analyses, and it was not possible to use inertial units because these could have resulted in discomfort for the participants, and inertial units are not a commodity among the public. Furthermore, we chose to place the FC4 on the wrist of the nondominant arm, as this placement is recurrent in daily life and research, although we knew that the movements would be inferior to those of the dominant wrist and therefore would reduce the artifact rate. Finally, the low number of participants included for the running activity (n=3) should be considered while interpreting our results, although the pooled analysis lowered this potential bias while providing similar reliability and accuracy results.

### Comparison With Previous Works

A recent study evaluated the accuracy of the FC4 HR sensor against an ECG Holter monitor for activities of daily living (sitting, walking, typing, lying down, etc) and showed acceptable HR measurement capabilities [47]. The added value of our protocol comes from the ecological approach to the gear sensor validation, which included additional physical activities with various intensities and limb movements. To our knowledge, no study has evaluated the FC4 in this manner during multiple sports or activities yet. However, further studies should be conducted to measure the reliability of the FC4 and its parameters for activities of daily living (number of steps, number of stairs climbed, number of calories burned, and quality of sleep), especially among persons or patients whose physical activities are restricted, as observed in sedentary individuals, people with obesity, people with heart failure, etc.

Considering our results (ie, the lack of precision and reliability in specific activities), the FC4 should not be used for research or athlete training purposes, including those related to running, which showed the lowest LOA width and artifact ratio. However, the FC4 could be useful for tracking HR during daily activities, which does not require such accurate monitoring, and it may be considered for patients’ reeducation. However, for the latter, further studies should inspect the FC4’s reliability according to the characteristics of the population. For example, obesity affects the physiological factors necessary for proper PPG signal intensity and quality, such as capillary density and recruitment, blood flow, and skin thickness [44].

### Conclusion

The FC4 shows excellent reliability for measuring HR during activities with slow and predictive arms movements, such as running. However, it should not be used for activities with “sharp” and random arm and wrist movements, such as soccer and racket sports, due to its sensitivity to motion artifacts. Hence, in ecological conditions, this device should not be used for research or training purposes due to the high artifact rate and LOA width.

## Abbreviations

bpm: beats per minute
CCC: Lin concordance correlation coefficient
ECG: electrocardiogram
FC4: Fitbit Charge 4
HR: heart rate
ICC: intraclass correlation coefficient
LOA: limit of agreement
MAE: mean absolute error
MAPE: mean absolute percentage error
PPG: photoplethysmography
